# Incidence and prevalence of eosinophilic oesophagitis: Are we reaching a plateau?

**DOI:** 10.1002/ueg2.12282

**Published:** 2022-08-15

**Authors:** Gwen M. C. Masclee, Albert J. Bredenoord

**Affiliations:** ^1^ Department of Gastroenterology and Hepatology Amsterdam UMC Amsterdam The Netherlands

Eosinophilic oesophagitis (EoE) can be defined as a chronic, local immune‐mediated oesophageal disease. EoE is characterized clinically by symptoms related to oesophageal dysfunction and histologically by eosinophil‐predominant inflammation. It is becoming clear that a key element for therapeutic management and prognosis includes early identification of the disease to avoid oesophageal fibrosis and strictures. From a previous cross sectional study on Spanish EoE patients we have learned that the time between start of symptoms and date of diagnosis of EoE has decreased over the past decade.^1^ Probably this improvement in EoE diagnosis will translate into better patient outcomes as reduced endoscopic severity at time of diagnosis should lead to better symptomatic and histological control and increased quality of life for patients.

EoE is becoming increasingly better recognized and becoming more common as well. Since its first description of EoE in 1993^2^ as a rare disease, it is now considered as one of the most prevalent oesophageal diseases. Many studies estimating the epidemiology of EoE have used selected samples. Studies using complete databases on nationwide rates of incidence and prevalence of EoE are scarce; firstly given the rarity of the disease and secondly the fact that a clear numerator (number of cases) and denominator (the background population) are needed. This means that regional or national information is required identifying all cases occurring in a certain time window, but also number of persons and time of living in this region—for example, information on date of death, and persons leaving the region is required. In Denmark, hospital data and pathology records are linked via a unique personal identification number. As such, data on both the number of cases and detailed background population are available. In the study by Hjøgaard Allin et al. standardized incidence rates for two definitions of EoE in Denmark between 2008 and 2018 are presented.^3^ The scale of the study having availability on nationwide data on clinical diagnosis codes (International Classification of Disease‐10) and pathology records is a unique feature of the study and provides excellent insights into the epidemiology of EoE. When only relying on pathology codes for EoE diagnosis (‘broad definition’) standardized incidence rates increased from 3.9 per 100,000 person‐years in 2011 to 11.7 per 100,000 person‐years in 2018. However, when taking symptoms of oesophageal dysfunction as a requirement as well for EoE diagnosis, standardized incidence rates were lower compared to the ‘broad definition’. This emphasizes two issues: (1) real life big scale data is hampered by the fact that it relies on coding and accuracy of the doctor that should have registered the code and that symptom data may not always be complete and (2) there may be a group of patients that may have undetected EoE as they do not have symptoms (yet). Whether the increase in incidence over time is a true rise or a perceived one by increased awareness by better adherence to guidelines,^4^ remains unclear. The latter can be investigated by dividing the number of cases by the number of gastroduodenoscopies with biopsies performed in the region during the same time period. Previously we have learned that for instance the initial rise in incidence of Barrett's oesophagus is not due to an increase in gastroduodenoscopies^5^ and for EoE it has been suggested by others that the increase in EoE incidence outpaces the rate of increase of biopsy rates,^6^ although data from these studies date back to early and mid‐2000s.^7^ In the current study, the authors note that the number of biopsies increased during the study period but the occurrence of EoE in biopsies remained stable, implying that the more widespread use of gastroduodenoscopies with biopsies may at least partially explain the increase. The findings of the study by Hjøgaard Allin et al. suggest that the detection work for gastroenterologists in the future remains important to identify all potential EoE patients.

Finally, while in clinical practice the differences between gastro‐oesophageal reflux disease (GORD) and EoE are usually clear due to the nature of symptoms and endoscopic features, it often remains challenging to separate GORD from EoE in epidemiological studies, as both diseases may have a degree of eosinophilia in biopsy specimens and may be registered as symptoms of oesophageal dysfunction.

## CONFLICT OF INTEREST

The authors have no conflicts of interest to declare.

## Data Availability

No data availability.
